# Plasma Lipoprotein-associated Phospholipase A2 in Patients with Metabolic Syndrome and Carotid Atherosclerosis

**DOI:** 10.1186/1476-511X-10-13

**Published:** 2011-01-19

**Authors:** Hui-ping Gong, Yi-meng Du, Li-na Zhong, Zhao-qiang Dong, Xin Wang, Yong-jun Mao, Qing-hua Lu

**Affiliations:** 1 Department of Cardiology, the Second hospital of Shandong University, Jinan, Shandong, 250033, China; 2 Department of Geriatric, affiliated hospital of medical college, Qingdao University, Qingdao, Shandong, 266003, China

## Abstract

**Background:**

Lipoprotein-associated phospholipase A_2 _(Lp-PLA_2_) is a recently identified and potentially useful plasma biomarker for cardiovascular and atherosclerotic diseases. However, the correlation between the Lp-PLA_2 _activity and carotid atherosclerosis remains poorly investigated in patients with metabolic syndrome (MetS). The present study aimed to evaluate the potential role of Lp-PLA_2 _as a comprehensive marker of metabolic syndrome in individuals with and without carotid atherosclerosis.

**Methods:**

We documented 118 consecutive patients with MetS and 70 age- and sex-matched healthy subjects served as controls. The patients were further divided into two groups: 39 with carotid plaques and 79 without carotid plaques to elucidate the influence of Lp-PLA_2 _on carotid atherosclerosis. The plasma Lp-PLA_2 _activity was measured by using ELISA method and carotid intimal-media thickness (IMT) was performed by ultrasound in all participants.

**Results:**

Lp-PLA_2 _activity was significantly increased in MetS subgroups when compared with controls, and was higher in patients with carotid plaques than those without plaques (*P *< 0.05). Furthermore, we found that significant difference in Lp-PLA_2 _was obtained between patients with three and four disorders of metabolic syndrome (*P *< 0.01). Age (β = 0.183, *P *= 0.029), LDL-cholesterol (β = 0.401, *P *= 0.000) and waist-hip ratio (β = 0.410, *P *= 0.000) emerged as significant and independent determinants of Lp-PLA_2 _activity. Multiple stepwise regression analysis revealed that LDL-cholesterol (β = 0.309, *P *= 0.000), systolic blood pressure (β = 0.322, *P *= 0.002) and age (β = 0.235, *P *= 0.007) significantly correlated with max IMT, and Lp-PLA_2 _was not an independent predictor for carotid IMT.

**Conclusions:**

Lp-PLA_2 _may be a modulating factor for carotid IMT via age and LDL-cholesterol, not independent predictor in the pathophysiological process of carotid atherosclerosis in patients with MetS.

## Background

The metabolic syndrome (MetS) is a constellation of atherogenic risk factors including abdominal obesity, hypertension, insulin resistance, dyslipidemia, proinflammatory, and prothrombotic state [1]. Recent publications have probed that patients with MetS are at higher risk of cardiovascular morbidity and mortality [2] and are more prone to atherosclerosis than normal subjects, even in the young adults [3,4]. Although the relationship between MetS and the risk of cardiovascular disease is still a matter of debate, MetS has been associated with carotid plaque formation and intima-media thickening [5]. Inflammatory processes have been increasingly recognized as a critical step in the pathogenesis of both metabolic syndrome and carotid atherosclerosis and may be important midways linking MetS to the increased arteriosclerotic events [6,7].

Lipoprotein-associated phospholipase A_2 _(Lp-PLA_2_) was recently characterized as a novel inflammatory biomarker correlated with several components constituting the MetS and implicated in atherosclerosis, incident cardiovascular disease [8,9]. Lp-PLA_2 _is preferentially secreted by monocytes and macrophages and hydrolyzes oxidatively modified low-density lipoprotein by cleaving oxidized phosphatidylcholines thereby generating lysophosphatidylcholine and oxidized free fatty acids [10]. Such chemoattractants are thought to play pivotal role in inflammatory reactions and particularly in vascular inflammation and atherosclerosis [11]. However, the potential role of Lp-PLA_2 _in atherogenesis and the anti- or proatherogenic characteristic of this enzyme in humans are less well understood [12]. Almost all prospective and nested case cohort studies suggested that Lp-PLA_2 _is proatherogenic [13]. One recent trial [14] demonstrated that symptomatic carotid artery plaques are characterized by increased levels of Lp-PLA_2 _and its product lysoPC in correlation with markers of tissue oxidative stress, inflammation, and instability. In contrast, previous investigations reported no associations observed between carotid intima-media thickness and Lp-PLA_2 _levels in primary hyperlipidemia patients [15,16].

To the best of our knowledge, few studies have explored the atherosclerotic risk for carotid arteries correlated with MetS is confounded by an association with activity of Lp-PLA_2_. Additionally, carotid intima-media thickness (IMT) of arteries is a useful measure of clinical atherosclerosis as assessed noninvasively by ultrasonography. Alternations in carotid IMT has been validated as a vascular marker of the progression of atherosclerosis [17]. Therefore, in the present study, we measured the plasma Lp-PLA_2 _activity in patients with MetS (including with and without carotid atherosclerosis) and correlated it with anthropometric parameters and carotid IMT to evaluate the possible contribution of Lp-PLA_2 _to carotid atherosclerosis.

## Methods

### Study Population

A total of 118 patients with MetS (53 men and 65 women, aged from 32 to71 years), were recruited from the Second Hospital of Shandong University according to the criteria proposed by the International Diabetes Federation [18]. Individuals were excluded if they had a clinical history of cerebrovascular disease or present neurological abnormalities, cerebral hemorrhage and severe cardio-renal or nutritional disorders, lipid and glucose metabolism. The control group consisted of 70 age- and sex-matched healthy subjects who visited our hospital for a routine physical check-up and without a history of cardiac disease, hypertension or diabetes and having normal findings on physical examination, chest roentgenography, and echocardiography. Informed consent was obtained from all participants and the study was approved by the local ethics committee.

### Definition of metabolic syndrome

In our study, metabolic syndrome was defined by the presence of 3 or more of the following conditions based on the criteria of IDF [18]: (1) visceral obesity: waist circumference was ≥ 90 cm in men and and ≥80 cm in women, (2) hypertriglycedemia: ≥ 150 mg/dl (1.7 mmol/l) or specific treatment for this lipid abnormality, (3) low HDL cholesterol: <40 mg/dl (1.03 mmol/l) in men and <50 mg/dl (1.29 mmol/l) in women or specific treatment for this lipid abnormality, (4) hypertension: systolic blood pressure ≥130 mmHg or diastolic blood pressure ≥85 mmHg or treatment of previously diagnosed hypertension, and (5) impaired fasting glucose concentration ≥100 mg/dl (5.6 mmol/l) or those who had been treated for type 2 diabetes.

### Clinical measurements

The baseline and clinical characteristics of all participants were determined. The details of age, gender and the weight and height were obtained, with the body mass index calculated as the body weight in kilogram divided by the height in meters squared. Waist circumference was measured at the level of the umbilicus, systolic and diastolic blood pressures were obtained with a mercury sphygmomanometer using auscultory methods.

The laboratory measurements were carried out following overnight fasting. Blood was collected at baseline for glucose, HbA1c, total cholesterol, triglycerides, high density lipoprotein (HDL)-cholesterol and low-density lipoprotein (LDL)-cholesterol. Serum insulin levels were determined by a radio-immunoassay kit (Dongya Ltd, Beijing, China). Insulin resistance was assessed by the homeostasis model assessment equation [19].

### Lp-PLA_2 _activity assay

The total plasma Lp-PLA_2 _activity was measured using a PAF Acetylhydrolase enzyme immunoassay (EIA) kit (Catalogue No. 760901, Cayman chemical Company , USA) with a lower limit of sensitivity of 0.02-0.2 umol/min/ml. Samples were measured in duplicate in a single experiment. Lp-PLA_2 _activity was expressed as micromoles of platelet-activating factor hydrolyzed per minute per milliliter of plasma samples and the inter-assay coefficient of variance was < 5%.

### Carotid ultrasonography

All participants were examined in the supine position (head turned 45°) by the same trained operator with a high resolution B-mode ultrasonography equipped with a 5-10 MHz linear array transducer (iE33, Philips Ultrasound, Washington, USA). ECG leads were attached to the ultrasound recorder for on-line continuous heart rate monitoring. All the images were recorded and stored on magneto optical disk for later playback and analysis. The right and left common carotid arteries (CCAs) and internal carotid arteries (including bifurcations) were evaluated. IMT, plaque extent of the near and far walls of the common and internal carotid arteries (ICAs) and bifurcations were measured according to the ACAPS protocol. AA thickened IMT was defined as ≥1.0 mm in either carotid artery. Presence of atherosclerotic plaques, defined as localized lesions with protrusion into the arterial lumen or regional IMT≥1.1 mm [20], was considered when found in either or both CCAs. IMT was therefore measured at the point of maximal thickness in the walls of both CCAs. Maximal and mean IMT were defined as the greatest and mean values, respectively, of IMT measured from 3 contiguous sites at 1-cm intervals. Maximal IMT represented the highest single measurement at any site with plaque. Both thickened IMT and plaques were reconfirmed by re-examining the lesions on the printouts from the ultrasound scanner.

### Statistical analysis

Data are presented as mean ± SD for continuous variables or proportions. After testing for normal distribution of variables, student's 2-tail t-test and one-way analysis of variance (anova) followed by the post hoc least significant difference test were used where appropriate. The correlations between two variables were assessed by Pearson correlation analysis. Multiple linear regression analysis was used to evaluate the contribution of independent factors. Statistical analyses were performed using SPSS v. 15.0 (SPSS, Chicago, IL) software. A two-tailed P value <0.05 was considered statistically significant.

## Results

### Baseline and clinical characteristics of participants

The baseline and clinical characteristics of the metabolic syndrome patients and controls were shown in Table 1. All MetS patients were divided into subgroups according to presence or absence of plaques, carotid atherosclerotic plaques was identified in 39 patients, and no stenosis or occlusion was found. There were no statistical differences in age or gender among three groups. Patients with MetS showed increased levels of systolic blood pressure, diastolic blood pressure, BMI, waist circumference, waist-hip ratio, triglyceride, total cholesterol, fasting glucose, insulin, HbA1c, HOMA-insulin resistance and more prescription of medications, and decreased levels of HDL-cholesterol when compared with controls (all *P *< 0.05-0.01), but there were no significant differences in any of those parameters between patients with and without carotid plaques. LDL cholesterol was found to increase from controls to MetS patients with and without carotid plaques, moreover, with significant difference between two patient subgroups (*P *< 0.05). Mean IMT values were significantly higher in MetS patients with and without carotid plaques than in controls (0.74 ± 0.11 mm vs. 0.51 ± 0.15 mm, 0.86 ± 0.20 mm vs. 0.51 ± 0.15 mm, all *P *< 0.01, respectively), and were highest in patients with carotid plaques. Max IMT values increased significantly in MetS patients with carotid plaques, whereas no difference was found between the other two groups.

**Table 1:** Baseline and clinical characteristics of the MetS subgroups and controls

Variables	Controls	MetS	
		No carotid plaque	With carotid plaque
Number	70	79	39
Sex (male/female)	30/40	35/44	18/21
Age (years)	52.4 ± 8.8	53.8 ± 7.8	55.4 ± 6.6
BMI (kg/m^2^)	24.49 ± 2.22	28.99 ± 4.05	28.07 ± 3.45
Waist circumference (cm)	84.51 ± 8.05	98.08 ± 10.16	96.45 ± 8.58
Waist-to-hip ratio	0.86 ± 0.05	0.92 ± 0.06	0.93 ± 0.08
Systolic BP (mmHg)	116 ± 10	151 ± 22	152 ± 23
Diastolic BP (mmHg)	77 ± 7	95 ± 14	94 ± 13
Total cholesterol (mmol/L)	4.42 ± 0.78	5.36 ± 1.12	5.55 ± 0.99
Triglyceride (mmol/L)	0.99 ± 0.42	2.34 ± 1.19	2.23 ± 0.94
HDL cholesterol (mmol/L)	1.53 ± 0.33	1.22 ± 0.31	1.25 ± 0.26
LDL cholesterol (mmol/L)	2.82 ± 0.72	3.63 ± 0.88**	4.03 ± 0.99**▲
Glucose (mmol/L)	4.99 ± 0.54	6.37 ± 2.09**	6.76 ± 3.01**
Insulin (uU/mL)	12.15 ± 5.37	21.39 ± 12.09**	19.63 ± 10.20**
HbA1c( % )	4.58 ± 0.32	5.38 ± 1.05	5.57 ± 1.52
HOMA-insulin resistance	2.74 ± 1.38	6.24 ± 4.90**	5.83 ± 3.45**
Mean IMT (mm)	0.51 ± 0.15	0.74 ± 0.11	0.86 ± 0.20▲▲
Max IMT (mm)	0.61 ± 0.52	0.77 ± 0.09	1.87 ± 0.73▲▲
Lp-PLA2 activity	24.14 ± 6.33	29.62 ± 8.98**	34.10 ± 9.51**▲▲
Medifications			
Aspirin	-	70(88%)	35(91%)
Anti-hypertensive drugs	-	30(38%)	17(43%)
Oral hypoglycaemic drugs	-	9(12%)	4(11%)
Lipid regulating agents	-	9(12%)	5(14%)

Values are mean ± SD; *P < 0.05 and **P < 0.01 compared to control; ▲*P *< 0.05 and ▲▲*P *< 0.01 compared to MetS without carotid plaque

### Lp-PLA_2 _activity

**Figure 1:**
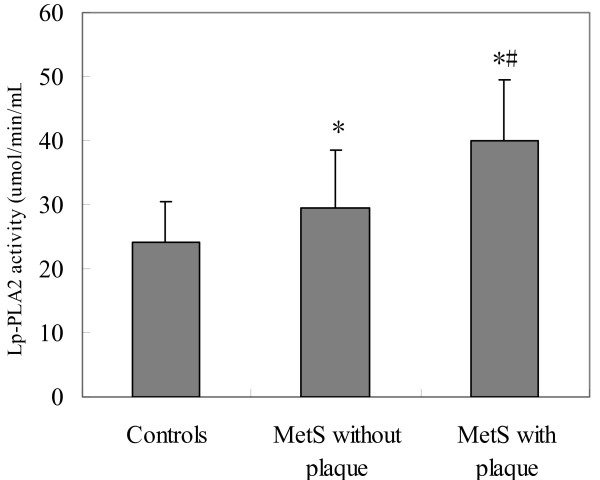
**Lp-PLA**_**2 **_**activity in the MetS subgroups and controls**.

Distribution of Lp-PLA_2 _activity approximates a normal distribution. Lp-PLA_2 _activity was significantly increased in MetS subgroups when compared with controls (all *P *< 0.01), and was higher in patients with carotid plaques than those without plaques (34.10 ± 9.51 umol/min/mL vs. 29.62 ± 8.98 umol/min/mL, *P *< 0.05) (Table 1, Figure 1). There were no age (<65 vs. ≥65 years) and gender differences of Lp-PLA_2 _activity in patients (Data not shown). To assess the association of metabolic syndrome components with Lp-PLA_2 _activity, we further found that significant difference in Lp-PLA_2 _was obtained between patients with three (n = 89) and four (n = 28) disorders of metabolic syndrome (38.79 ± 9.22 umol/min/mL vs. 30.60 ± 9.58 umol/min/mL, *P *< 0.01).

### Relationship of Lp-PLA_2 _activity and determinant factors

In simple regression analyses, Lp-PLA_2 _activity correlated positively with age (r = 0.250, *P *= 0.006), total cholesterol (r = 0.371, *P *= 0.000), LDL-cholesterol (r = 0.402, *P *= 0.000), glucose (r = 0.188, *P *= 0.042) and HbA1c (r = 0.188, *P *= 0.042) in the patients with MetS. We also found Lp-PLA_2 _activity correlated with waist-hip ratio weakly but not significantly (r = 0.174, *P *= 0.061). However, in multivariable stepwise regression analyses, age (β = 0.183, *P *= 0.029), LDL-cholesterol (β = 0.401, *P *= 0.000) and waist-hip ratio (β = 0.410, *P *= 0.000) emerged as significant and independent determinants of Lp-PLA_2 _activity (Table 2).

**Table 2:** Linear regression analysis of variables correlated with Lp-PLA2 in Mets patients

	Simple		Multiple	
	r	P	β	P
Age (years)	0.250	0.006	0.183	0.029
Sex	0.164	0.076	0.185	0.073
BMI (kg/m2)	-0.092	0.326	0.148	0.270
Waist-to-hip ratio	0.174	0.061	0.410	0.000*
Systolic BP (mmHg)	-0.081	0.386	-0.068	0.407
Diastolic BP (mmHg)	-0.150	0.106	-0.065	0.435
Total cholesterol (mmol/L)	0.371	0.000	0.065	0.618
Triglyceride (mmol/L)	0.082	0.380	0.065	0.791
HDL cholesterol (mmol/L)	-0.025	0.786	-0.071	0.407
LDL cholesterol (mmol/L)	0.402	0.000	0.401	0.000*
Glucose (mmol/L)	0.188	0.042	0.084	0.305
Insulin (uU/mL)	-0.153	0.099	-0.122	0.142
HbA1c	0.188	0.042	0.084	0.305
HOMA-insulin resistance	-0.069	0.458	-0.084	0.311

### Relationship of max IMT and risk factors

Pearson's correlation coefficient and multiple regression analysis were performed to examine the relationship of max IMT to Lp-PLA_2 _activity and other biomarkers in overall MetS patients in order to identify a parameter that reflects carotid atherosclerosis. As shown in Table 3, Lp-PLA_2 _activity (r = 0.199, *P *= 0.023), LDL-cholesterol (r = 0.333, *P *= 0.000), age (r = 0.325, *P *= 0.000) and systolic blood pressure (r = 0.225, *P *= 0.015) were significantly correlated with max carotid IMT. Unexpectedly, in multiple stepwise regression analysis, Lp-PLA_2 _activity correlated with the presence of atherosclerosis weakly but not significantly (β = 0.146, *P *= 0.097). Our results revealed that only LDL-cholesterol (β = 0.309, *P *= 0.000), systolic blood pressure (β = 0.322, *P *= 0.002) and age (β = 0.235, *P *= 0.007) were the significant predictors of max IMT. Moreover, after adjustment for age and lipid variables, the associations between Lp-PLA_2 _activity and carotid IMT did not reach statistical significance.

**Table 3:** Linear regression analysis of risk factors correlated with max-IMT in Mets patients

	Simple		Multiple	
	r	P	β	P
Age (years)	0.325	0.000	0.235	0.007
Sex	0.011	0.907	0.226	0.112
BMI (kg/m2)	0.128	0.245	0.092	0.274
Waist-to-hip ratio	0.071	0.449	0.016	0.874
Systolic BP (mmHg)	0.225	0.015	0.322	0.002
Diastolic BP (mmHg)	0.108	0.245	0.097	0.285
Total cholesterol (mmol/L)	0.119	0.200	0.065	0.541
Triglyceride (mmol/L)	0.052	0.573	0.013	0.882
HDL cholesterol (mmol/L)	-0.039	0.676	-0.008	0.927
LDL cholesterol (mmol/L)	0.333	0.000	0.309	0.000
Glucose (mmol/L)	0.113	0.225	0.104	0.201
HbA1c	0.113	0.225	0.104	0.201
HOMA-insulin resistance	0.007	0.940	0.011	0.893
Lp-PLA2	0.199	0.023	0.146	0.097

## Discussion

The present study identified elevated total plasma Lp-PLA_2 _activity in patients with the MetS, especially in those with carotid atherosclerosis when compared to the control subjects. We demonstrated that the Lp-PLA_2 _activity correlated with age, LDL-cholesterol and waist-hip ratio in patients with metabolic syndrome. Unfortunately, our data provided no evidence that Lp-PLA_2 _activity independently influence carotid IMT in MetS patients. The associations of Lp-PLA_2 _activity with carotid atherosclerosis may be mediated through age and LDL-cholesterol level.

The biological mechanisms involving plasma Lp-PLA_2 _in the pathogenesis of the MetS and atherosclerosis are not well-characterized. Recent evidence suggests inflammation is an important pathogenic factor in atherosclerosis and coronary heart disease, particularly in the context of insulin resistance, obesity [21], and the metabolic syndrome [6]. Furthermore, atherosclerosis is now recognized as manifestations of vascular inflammation [7]. Inflammatory factors such as adhesion molecules (ICAM-1 and VCAM-1), CD40 ligands, C-reactive protein (CRP) and myeloperoxidase (MPO) participate in induction of insulin resistance and atherosclerotic disease [21,22]. Lp-PLA_2_, originally named platelet-activating factor acetylhydrolase (PAF-AH), is an enzyme involved in lipoprotein metabolism and inflammatory pathways [10]. In human, 80% of Lp-PLA_2 _circulates bound to LDL- cholesterol, 10-15% circulates with HDL-cholesterol, and the remaining 5-10% circulates with VLDL-cholesterol [18]. Lp-PLA_2 _enzymatic activity results in generation of lysophosphotidylcholine (lysoPC) and oxidized non-esterified fatty acids, two pro-inflammatory mediators [10]. The lysoPC stimulates macrophage proliferation, up-regulates cytokines and CD40 ligands, and increases the expression of vascular adhesion molecules, implying a complex interaction between Lp-PLA_2 _and other inflammatory mediators [23,24]. Based on that, Lp-PLA_2 _has been implicated in inflammation and considered as an inflammatory marker in the MetS. Recently, several epidemiological studies demonstrate that an elevated activity of Lp-PLA_2 _is associated with MetS and number of the metabolic syndrome components as well as incident fatal and non-fatal CVD regarding MetS [25,26]. In the current study, we observed that plasma Lp-PLA_2 _activity was higher in patients with the MetS than in controls, suggesting that Lp-PLA_2 _activity may increase significantly when metabolic syndrome was present. Furthermore, our findings showed that there was a linear rise in Lp-PLA_2 _activity with an increment of number of metabolic syndrome components. These data were in line with previous research [19] and enlarged our scope for potential role of Lp-PLA_2 _in patients with MetS.

Results for studies of the associations of components of MetS with Lp-PLA_2 _activity have shown that abdominal obesity may have been independently responsible for the changes of Lp-PLA_2 _observed in this study. Additionally, we found Lp-PLA_2 _correlated with glucose and HbA1c weakly but significantly only in simple regression. Our findings were in accordance with the previous studies, in which Lp-PLA_2 _correlated with abdominal adiposity [27,28] but differed from the results of Noto et al [25] and Rana et al [29], who found that plasma Lp-PLA_2 _activity did not appear to be associated with waist circumference. It was possible that obesity was associated with decreases in local and peripheral insulin resistance [30]. Adipose tissue located in intra- abdominal or visceral cavities is likely to be infiltrated by macrophage, which is an important cause of the inflammatory state associated with abdominal obesity and the metabolic syndrome [29]. Lp-PLA_2_, as an inflammatory marker, is mainly secreted by macrophages. Thus, our results suggested that central obesity may contribute to the Lp-PLA_2 _activity changes in patients with MetS. Out results also indicated that there was parallel increase in Lp-PLA_2 _activity with an increment of components of metabolic syndrome, which was consistent with the findings of Noto at al. [25].

In the present study, our results have shown that Lp-PLA_2 _activity was elevated among MetS patients with carotid plaques. However, we further found independent determinants for thickened IMT as being LDL-cholesterol and age in multiple regression models. The Lp-PLA_2 _activity was not independently facilitates the morbidity of carotid atherosclerosis. Previous studies have revealed the plasma Lp-PLA_2 _activity in atherosclerotic disease, but consensus is still lacking [8,9,31]. Biologically, Lp-PLA_2 _is a vascular-specific proinflammatory enzyme that operates physiologically in the arterial intima [32]. Evidence has shown that Lp-PLA_2 _is expressed in human and rabbit atherosclerotic plaques [33]. Vickers et al [34] revealed that carotid tissue concentrations of Lp-PLA_2 _was notably very high in the rupture-prone shoulder region of thin fibrous cap atheromas, and Lp-PLA_2 _colocalized with macrophages and oxidized LDL in atherosclerotic carotid plaques. However, several clinical studies suggested that premature coronary atherosclerosis [31] as well as carotid intima-media thickness plasma was not influenced by Lp-PLA_2 _activity and gene polymorphisms in hypercholesterolemic individuals [15,16]. Thus, consistent with results of previous study [35], role of this enzyme in predicting independently the thicken IMT attenuated in our study, especially after adjustment for age and lipid variables.

Although the exact mechanisms underlying contribution of Lp-PLA_2 _to carotid atherosclerosis in MetS patients remain to be elucidated, there are several possible explanations. Firstly, our study demonstrated that lipid parameters may contribute to Lp-PLA_2 _activity changes. An increase in plasma Lp-PLA_2 _activity, reflecting LDL-cholesterol values, has been established in several investigations [9,36]. Stafforini et al investigated that Lp-PLA_2 _participated in the key oxidative steps of atherogenesis due to the association of Lp-PLA_2 _and LDL-cholesterol via an interaction with apolipoprotein B (apoB) [37]. Kawamoto et al [38] reported that LDL-cholesterol was independently associated with carotid atherosclerosis in addition to clustering of cardiovascular risk factors regarding MetS. Several studies [39-41] suggested that the components of metabolic and LDL-cholesterol played a role to synergistically influence vascular thickness. Our results revealed that levels of LDL-cholesterol were significantly increased in MetS patients with carotid plaques than those without. Secondly, in present study, multivariate linear regression showed that age had a similar positive association with Lp-PLA_2 _activity and contributed strongly to the variation in IMT. Thus, taken together previous important results [38-41] and our intriguing findings implied that Lp-PLA_2 _activity was intimately associated with carotid thicken IMT and atherosclerosis via correlation with age and LDL-cholesterol in our study. Lp-PLA_2 _may be a modulating factor in the process of carotid atherosclerosis. Lastly, owing to the prominent biological activities, the opposing proinflammatory and antiatherogenic properties of Lp-PLA_2 _have been demonstrated both in human and animal models. In rabbit models, administration of Lp-PLA_2 _inhibited myocardial ischemia/reperfusion injury [42], and local expression of Lp-PLA_2 _reduced accumulation of oxidized LDL-cholesterol in balloon-injured arteries [43]. However, previous findings did not ascertain a causal relationship between Lp-PLA_2 _and the clinical consequences of atherosclerotic disease in patients with primary hyperlipidemia [15,16] and diabetes mellitus [44]. Interestingly, elevated Lp-PLA_2 _has been identified in human symptomatic carotid atherosclerotic plaque and its product lysophosphatidylcholine (lysoPC) correlated with markers of tissue oxidative stress, inflammation, and instability [14]. These paradoxical results may partly be explained by the relatively small study populations and selected inclusion criteria. Further studies are required to clarify whether Lp-PLA_2 _is a risk marker that participates in the pathogenesis of carotid atherosclerosis in patients with MetS.

Despite of interesting findings, potential limitations of this study merit consideration. Our results are based on single measurements of circulating Lp-PLA_2_, which may not reflect the true activity of Lp-PLA_2 _over time or true expression in carotid atherosclerotic plaques. For this reason, further outcome-directed prospective studies would give insight into the significance of Lp-PLA_2 _activity versus expression in atherosclerotic plaques. Several studies have shown that activity of Lp-PLA_2 _was impacted by lipid-lowering drugs such as statins and fibric acid derivatives (fibrates)[25,45]. Thus, we could not eliminate the possible effect of medications for Lp-PLA_2 _activity on the present findings. Finally, even though intriguing, results obtained in further confirmatory studies need to be considered to clarify the validity of Lp-PLA_2 _in large series of patients.

## Conclusions

In conclusion, our results of increased plasma Lp-PLA_2 _activity in patients with the metabolic syndrome, especially in those with carotid atherosclerosis, suggest that Lp-PLA_2 _may be an inflammatory marker of metabolic syndrome. However, multiple stepwise regression analysis suggested that Lp-PLA_2 _may be a modulating factor, not independent risk predictor in the pathophysiological process of carotid atherosclerosis in MetS patients. Because Lp-PLA_2 _activity may represent a novel pathway associated with thicken IMT, further research using large samples and general population need to be done to clarify the exact role of Lp-PLA_2 _on carotid atherosclerosis in metabolic syndrome subjects.

## Competing interests

The authors declare that they have no competing interests.

## Authors' contributions

HPG and YMD participated in the design of the study and drafted the manuscript; ZQD and LNZ performed research; HPG and XW analyzed data; YJM and QHL were responsible for the study design and the funding. All authors read and approved the final manuscript.
